# Decitabine‐Driven Foetal Haemoglobin Induction in Townes Mice and Human Erythroblasts

**DOI:** 10.1002/jha2.70120

**Published:** 2025-08-04

**Authors:** Ariadna Carol Illa, Desmond Wai Loon Chin, Martha Clark, Jesper Petersen, Søren Skov, Andreas Glenthøj, Carsten Dan Ley

**Affiliations:** ^1^ Rare Disease Research Global Drug Discovery Novo Nordisk A/S Måløv Denmark; ^2^ Department of Veterinary and Animal Sciences Faculty of Health and Medical Sciences University of Copenhagen Copenhagen Denmark; ^3^ Rare Disease Research Global Drug Discovery Novo Nordisk Research & Development US, Inc. Boston Massachusetts USA; ^4^ Danish Red Blood Cell Center Department of Hematology Copenhagen University Hospital – Rigshospitalet Copenhagen Denmark; ^5^ Department of Clinical Medicine University of Copenhagen Copenhagen Denmark

**Keywords:** haemoglobin disorders, haemoglobinopathies, haemolysis, red blood cell disorders, red cell disorders, sickle cell disease, sickle cell anaemia

## Abstract

**Background:**

Induction of foetal haemoglobin (HbF) is a clinically validated approach to modulate the severity of sickle cell disease (SCD). This manuscript evaluates the efficacy of decitabine, a DNA methyltransferase (DNMT) inhibitor, in inducing HbF in healthy human erythroblasts and Townes mice, which are well‐established systems modelling SCD.

**Methods:**

Healthy human erythroblasts were treated with decitabine, and HbF induction was measured. Townes sickle cell mice were administered decitabine for 12 weeks, and various haematological parameters were assessed.

**Results:**

In healthy human erythroblasts, decitabine treatment resulted in a significant increase in the fraction of HbF‐rich cells (F‐cells), accompanied by elevated HbF protein levels. The HbF induction was superior to that achieved with hydroxyurea, the primary therapy for SCD. In Townes mice, the maximal response was observed after 12 weeks of dosing, with an increase in both HbF protein and F‐cells, alongside reduced red blood cell and reticulocyte counts. Additionally, we observed changes in other haematological parameters, such as increased mean corpuscular volume and mean corpuscular haemoglobin. However, the HbF induction observed in the mice was modest relative to known human responses. No marked improvements in SCD‐related biomarkers such as haemolysis or liver function were detected, suggesting that the mouse model may not fully capture the extent of phenotype improvement. Histopathological examination revealed no adverse effects on bone marrow cellularity or morphology and indicated a protective effect on liver tissue integrity.

**Conclusion:**

Our results demonstrate that decitabine induces HbF in a dose‐dependent manner in both in vitro and in vivo settings, highlighting the complexity of HbF induction as a treatment for SCD and underscoring the need for further refinement of this model for SCD therapy research.

**Trial Registration**: The authors have confirmed clinical trial registration is not needed for this submission.

## 1 Introduction

Sickle cell disease (SCD) is an inherited disease caused by a single‐nucleotide mutation in the gene encoding the β‐globin subunit of haemoglobin (Hb). This mutation leads to the production of sickle haemoglobin (HbS), which is prone to polymerize under hypoxic conditions, resulting in the deformation (i.e., sickling) of red blood cells (RBCs), increased haemolysis, and progressive organ damage [1]. Vaso‐occlusive crises (VOCs), characterized by the blockage of small blood vessels by aggregates of sickled RBCs, white blood cells (WBCs) and platelets (PLTs), are the hallmark of SCD and can trigger acute, painful episodes, leading to tissue ischaemia and infarction [2]. These crises are a major cause of morbidity and mortality associated with SCD and can affect multiple organ systems, including the spleen, brain, lungs and kidneys [3].

Despite recent regulatory approval of *ex vivo* gene therapy for SCD, curative options remain unavailable to most patients, and the mainstay of treatment globally is hydroxyurea (HU). HU is orally administered and primarily works by inducing foetal haemoglobin (HbF). The consequent reduction in HbS not only decreases the likelihood of polymerization at lower oxygen tensions but also enhances the overall deformability of RBCs, thereby mitigating the risk of sickling and subsequent VOCs [4, 5, 6]. Recent years have seen the development of new treatment options for SCD [7, 8, 9]; however, a significant unmet need for novel oral treatments remains.

HbF consists of two α‐globin and two γ‐globin chains and has a higher affinity for oxygen compared to adult haemoglobin (HbA), which is composed of two α‐globin and two β‐globin chains. Importantly, the benefits of HbF induction extend beyond mere dilution effects on HbS concentration. RBCs containing HbF are less likely to become completely oxygen‐depleted [10]. Additionally, the unique structure of the γ‐globin chain offers anti‐sickling properties beyond those of normal β‐globin due to specific amino acid changes, preventing polymerization even when deoxygenated [11, 12]. Elevated levels of HbF in adults with SCD have been associated with a reduced risk of disease‐related complications, including RBC sickling, VOCs and haemolysis, making induction of HbF an attractive strategy for therapeutic intervention [1, 11, 13, 14, 15, 16]. Clinical studies have demonstrated the beneficial effects of HbF induction in managing SCD [4, 17, 18, 19].

After birth, there is an epigenetic shift from the predominant HbF to HbA or HbS in SCD, resulting in low HbF expression levels [10, 11]. This shift involves the suppression of γ‐globin genes and the activation of β‐globin genes by a combination of transcriptional and epigenetic modifications, including methylation of gene promoter regions [20].

DNA methyltransferase 1 (DNMT1) is an important enzyme responsible for maintaining DNA methylation at CpG sites and is known to be pivotal in regulating HbF expression [21]. Decitabine (5‐aza‐2ʹ‐deoxycytidine), a deoxycytidine analogue initially developed for the treatment of myelodysplastic syndromes, acts as a prodrug, which must be converted into a nucleoside triphosphate to be active [22, 23, 24]. Once activated, it incorporates into the DNA of dividing cells during the S‐phase of the cell cycle [22]. The presence of decitabine in the nascent DNA during cell replication results in the formation of an irreversible covalent bond with the catalytic site of DNMT1, inducing ubiquitin‐E3 ligase activity and subsequent proteasomal degradation of the enzyme, ultimately inhibiting DNA methylation [25]. Similar properties are shared by other cytidine analogues, such as 5‐azacytidine [26, 27].

Therefore, by inhibiting DNA methylation, there is an opportunity to re‐activate γ‐globin gene expression, leading to increased HbF production, potentially mitigating the adverse effects of SCD [28]. Several preclinical studies have demonstrated a positive effect of decitabine on HbF induction in non‐human primates [29, 30], and there is clinical evidence supporting this mechanism [31, 32, 33, 34, 35]. However, there is a notable scarcity of preclinical publications using mouse models of SCD treated with cytidine analogues such as decitabine, alone or in combination with cytidine deaminase (CDA) inhibitors (CDA is known to inactivate cytidine analogues, limiting their therapeutic efficacy) [24, 36]. The limited available data includes a single 12‐day study in Townes mice, which reported an increase in HbF levels within this short timeframe [37]. This response is notably earlier compared to the 4 weeks required to observe similar effects in humans [34]. Given the scarcity of such studies, further research is needed to validate these findings in mouse models, as the current evidence for the translational efficacy of decitabine between mouse models and human SCD patients is very limited.

Preclinical models in vitro, *ex vivo* and in vivo play a pivotal role in the study of SCD, offering vital insights into the pathophysiology and efficacy of potential treatments. in vitro models using human cells, such as erythroid progenitor cells derived from patients with SCD or healthy donors, allow for the examination of specific cellular responses, such as the induction of HbF [38, 39, 40] and the decitabine‐dependent depletion of DNMT1 [41]. Wild‐type animals, though not carrying the SCD mutation, can be useful for comparative studies, particularly in understanding baseline haematologic function and response to stressors. However, mice and humans exhibit distinct globin gene clusters. Mice only have embryonic (ɛ)‐specific Hbs (ɛY, βh1) and HbAs (β1 and β2), while humans have foetal‐specific Hb genes (Gγ, Aγ) besides their ɛ and adult genes (δ and β). These differences, related to the β‐globin subunit of Hb, complicate direct comparisons of gene regulation between wild‐type mice and humans [42, 43].

SCD‐specific mouse models, including genetically modified mice that express human HbS, may allow for a more comprehensive understanding of disease mechanisms and therapeutic testing, such as HbF induction, the study of *ex vivo* sickling and the study of systemic disease complications [44, 45, 46]. Among the most extensively studied SCD models is the Townes mouse model, where murine globin genes are replaced with their human sickle counterparts *(* *HBA1*, *HBB^s^* and *HBG1)*, including some proximal regulatory elements, although certain key DNA‐regulatory elements are absent [46, 47]. Unlike other SCD mouse models, Townes mice mimic the foetal‐to‐adult conversion that occurs in humans after birth and are thus protected by HbF during ɛ development and birth [10, 43, 46, 48, 49]. Starting at an early age, however, these mice exhibit pathological features similar to those observed in patients with SCD, such as anaemia, RBC sickling, altered nociception and multi‐organ dysfunction [49, 50, 51, 52, 53].

This study investigates the efficacy of decitabine in inducing HbF in both healthy human erythroblasts and Townes mice in order to explore the efficacy and translatability of HbF induction across models and evaluate the utility of decitabine as a benchmark compound for HbF induction studies.

## 2 Materials and Methods

### 2.1 CD34^+^ Cell 2‐Phase Erythroid Differentiation

Mobilized peripheral blood CD34^+^ cells from healthy donors (Allcells) were thawed, washed, and counted. A cell stock was prepared in Phase 1 medium containing X‐Vivo‐15 (#02‐060‐Q, Lonza), rhIL‐3 at 10 ng/mL (#200‐03, Peprotech), rhSCF at 100 ng/mL (#300‐07, Peprotech) and rhFlt‐3 ligand at 100 ng/mL (#300‐19, Peprotech). 40,000 cells/mL and cultured in a humidified incubator at 37°C, 5% CO_2_ for 7 days. On Day 7, the cells were washed with X‐Vivo media and then resuspended in a new medium containing X‐Vivo‐15, rhIL‐3 at 10 ng/mL, rhSCF at 100 ng/mL and Epo at 3 U/mL (#287‐TC, Bio‐techne). DMSO, HU (#8627‐1G, Sigma Aldrich) and decitabine (#2624, Bio‐techne) were spotted into 96‐well plates using an Echo liquid handler (Beckman Coulter). 250,000/mL cells were seeded in each well. Each test condition was set up in triplicate. The plates were returned to the incubator for an additional 7 days. Following incubation, the induction of HbF was assessed by flow cytometry or high‐performance liquid chromatography (HPLC).

### 2.2 HbF Flow Cytometry and HPLC for CD34^+^ Differentiated Cells

Cells were blocked with FC block solution (#60041, StemCell Technologies) at 4°C for 15 min. Following blocking, the surface markers of the cells were stained with an antibody cocktail containing CD235a‐PE/Cy7 (#349112, BioLegend) and CD71‐APC (#130‐115‐030, Miltenyi Biotec) at 4°C for 30 min. Next, the cells were stained with 1× fixable live/dead cell stain BV405 (#l34964, Life Technologies) at room temperature for 30 min. The cells were then washed with PBS, resuspended in 1× Cytofix/Cytoperm solution (#554714, BD Biosciences), and fixed for 20 min at 4°C. Post‐fixation, cells were washed twice with Perm/Wash Buffer, resuspended in 100 µL per well of Perm/Wash Buffer, and stained with anti‐HbF‐PE (#MHFH044, Life Technologies) for 30 min at room temperature. Finally, the cells were washed twice with Perm/Wash Buffer, resuspended in 100 µL per well of PBS + 2% FBS, and analysed using a Novocyte or MACSQuant 10 cytometer. F‐cells, defined as cells positive for anti‐HbF‐PE and thus containing some level of HbF, were quantified as a percentage of total cells.

Human Hb variants (HbF, HbA) were quantified by cation‐exchange HPLC on a Dionex Ultimate 3000 UHPLC System (Thermo Fisher) at Spectrus LLC (Beverly, MA). Protein HbF levels were plotted as a fraction of total Hb variants.

### 2.3 Immunoblot Assay

Cells were lysed in 5× packed volume of 1× RIPA buffer (#89900, Thermo Fisher Scientific) supplemented with HALT protease/phosphatase inhibitor (#1861281, Thermo Fisher Scientific) and Turbonuclease (#T4330, Sigma Aldrich). The pellets were sonicated. Protein concentration was estimated using the BCA protein assay reagent (#22662, Thermo Fisher Scientific). Equal amounts of protein were loaded and resolved using NuPage 4%–12% Bis‐Tris Midi gel (#WG1402BOX, Invitrogen). The membrane was blocked using PBS with 5% BSA (#A9418, Sigma‐Aldrich) and 0.1% Tween‐20 (#WG327634, Thermo Fisher Scientific). Antibodies against DNMT1 (#24206‐1‐AP, 1:1000, ProteinTech), BCL11A (#75432, 1:4000, Cell Signaling Technology), and Vinculin (#V9264, 1:10000, Sigma Aldrich).

### 2.4 Mice

Townes mice (JAX stock #013071) aged 3–4 weeks were procured from Jackson Laboratories, Bar Harbour, USA [46]. Male and female Townes mice were group‐housed in environmentally enriched cages under standardized conditions (21°C, 60% relative humidity, a 12‐h/12‐h light/dark cycle, and ad libitum access to food and water). Six mice, three male and three female, were randomized to each treatment group. The mice, aged 4–5 weeks, received subcutaneous doses of research‐grade decitabine (0.4 or 0.6 mg/kg; 10 mg #2624, Bio‐techne) or vehicle (MiliQ water) three times a week (M, W, F) for 12 weeks, and their weight was recorded three times a week. Experiments were approved and performed according to guidelines by the Novo Nordisk Ethical Review Council and the Danish Animal Experiments Inspectorate, the Ministry of Food, Agriculture, and Fisheries of Denmark.

The sample size of six mice was determined based on the mean and standard deviation (SD) of RBC counts from published data by Jackson Laboratory [54]. Assuming a two‐sided significance level of 0.05, this sample size provides 86% power to detect a biologically relevant 20% change (InVivoStat v4.9 [55]).

### 2.5 Peripheral Blood Samples

Blood was collected at the beginning of the study (Day 1), mid‐way (Week 6) and at study termination (Week 12). Mice were anaesthetized with isoflurane (induction at 5% isoflurane and maintenance anaesthesia at 2% [70% O_2_ and 30%N_2_O]) and blood was collected from the retrobulbar venous plexus using a capillary tube (25 µL EDTA #78213, Vitrex) into an EDTA‐coated Eppendorf tube (2mg/mL EDTA‐K_2_ #041‐TOM‐14C, Milian) for haematological analysis. Serum samples were collected with a non‐coated capillary (25 µL end‐to‐end plain #174613, Vitrex) into a tube for serum collection (#20.1290 Microvette 200, Sarstedt).

### 2.6 Complete Blood Counts

A 25 µL of EDTA‐blood was diluted into 100 µL of CELLPACK buffer (#834‐0011‐10, Sysmex) and processed within the subsequent 4 h using a Sysmex XT‐2000i Haematology Analyser (Kobe, Japan) in ‘Capillary mode’.

The following parameters were obtained from each sample: RBC count, Hb, absolute reticulocyte count, mean corpuscular volume (MCV), mean corpuscular haemoglobin (MCH), mean corpuscular haemoglobin concentration (MCHC), PLT counts, WBC count, haematocrit and red cell distribution width (RDW‐SD).

### 2.7 Metabolic Analysis

Samples collected for serum extraction were left to clot at room temperature for 30 min and then centrifuged at 4600 rpm for 10 min at 4°C. 40 µL of the serum layer was transferred to COBAS tubes and stored at −80°C until analysis. The samples were analysed using a COBAS 6000 c501 (Roche Diagnostics A/S) to measure lactate dehydrogenase (LDH).

### 2.8 HbF Flow Cytometry and HPLC on Peripheral Blood Samples

The percentage of F‐cells was determined by flow cytometry with a NovoCyte Quanteon (Agilent) using a mouse monoclonal anti‐human HbF antibody, allophycocyanin (APC) conjugated (Clone: HBF‐1, #MHFH05, Invitrogen) and an APC‐conjugated mouse IgG1 kappa isotype control (Clone: P3.6.2.8.1, #17‐4714‐82, eBioscience, Invitrogen), both at 0.83 µg/mL. Flow cytometry data were analysed with NovoExpress software (Agilent).

The percentage of HbF protein levels was determined by HPLC using the Variant II Hemoglobin Testing System (Bio‐Rad).

### 2.9 Decitabine Exposure

EDTA‐blood was centrifuged at 1300 g for 10 min at 4°C, and 50 µL of plasma was aliquoted into polypropylene tubes, pre‐spiked with 50 µL of 10 µg/mL of zebularine (#Z4775‐25MG, Sigma‐Aldrich) and frozen at ‐80°C until analysis. Analysis was done at Worldwide Clinical Trials (Austin, USA) on a liquid chromatography‐tandem mass spectrometry system (Sciex API 4000 LC‐MS‐MS) equipped with an HPLC column. Briefly, the peak areas of the decitabine and tetrahydrouridine product ions were measured against their respective internal standards, decitabine‐^15^N_4_ and tetrahydrouridine‐D_3_. Quantitation was achieved using weighted linear least squares regression analyses generated from calibration standards prepared on the day of extraction.

### 2.10 Tissue Sampling and Processing

After terminal blood sampling, mice were euthanized by exsanguination through an incision of the abdominal aorta. The following organs were extracted and weighed: spleen, liver, and sternum. All organs were fixed in 10% neutral buffered formalin (VWR International AB, Sweden) for 24–48 h. In addition, the sternum was decalcified for 15 days. All organs were dehydrated via graded concentrations of ethanol (Kemetyl A/S, Denmark), transferred to xylene (Applichem, GmbH, Germany) and embedded in paraffin; the sternum was decalcified before embedding.

### 2.11 Histochemistry and Image Analysis

Sections of the liver and sternum of 3 µm thickness were cut and stained with haematoxylin and eosin (H&E) (#MSH80‐2.5 L and #HT110208‐2.5 L; both from Sigma‐Aldrich, USA). A digital whole‐slide image of each liver and sternum section was generated by scanning the sections on a NanoZoomer 2.0HT digital slide scanner (Hamamatsu).

### 2.12 Quantification of Liver Tissue Types With HALO AI Image Analysis Software

The quantification of liver tissue sections' whole‐slide images was analysed using HALO AI 3.5 (Indica Labs) with two AI algorithms, as previously described [53]. The first algorithm, a Random Forest‐based model, identified tissue areas, while the second DenseNet V2 classifier categorized different tissue types (background, normal healthy liver tissue, RBC clot, necrotic tissue and diseased tissue). The classifications were validated by an experienced pathologist.

### 2.13 Statistical Analysis

Data are presented as individual mice for the in vivo data, with bars representing the mean ± SD. Statistical analysis was conducted using the statistical software R (packages ‘afex’, ‘emmeans’, ‘multcomp’, ‘sandwich’) [56].

For comparisons between treatment groups with a single time point, a one‐way heteroscedastic ANOVA was used. *p* values and 95% confidence intervals were adjusted for multiple testing using Dunnett's method.

For the assessment of treatment effects across multiple weeks within mice, a repeated measures ANOVA was conducted to account for correlated observations over time. Mauchly's test was utilized to check the assumption of sphericity, and when necessary, corrected *p* values were calculated using Greenhouse–Geisser and Huynh–Feldt corrections. Pairwise comparisons were performed with Tukey adjustment to account for multiple testing.

## 3 Results

### 3.1 Decitabine Induces HbF in Differentiated Primary Human CD34^+^ Erythroid Cells in vitro

**FIGURE 1:**
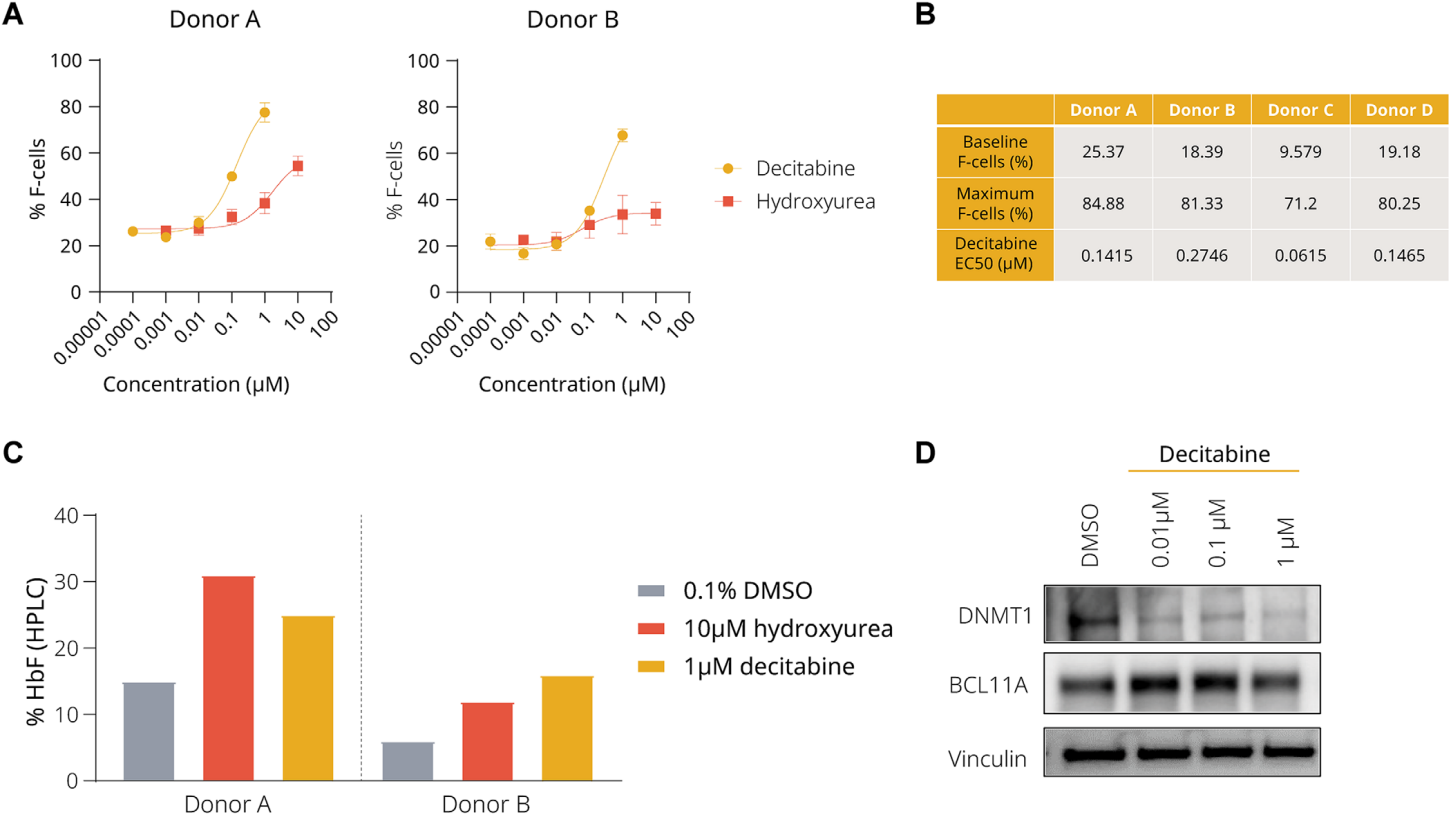
In vitro foetal haemoglobin (HbF) induction with decitabine. (A) Representative dose‐response curves of decitabine and hydroxyurea in two healthy donors. Data points for 3 µM decitabine and 30 µM hydroxyurea were excluded due to high cytotoxicity. Mean ± SD of technical triplicates are shown. (B) Baseline F‐cells, maximum F‐cells at 1 µM decitabine, and EC50 (half maximal effective concentration) of four healthy donors. (C) HbF protein levels by HPLC of two donors. (D) Immunoblot showing the expression of DNMT1 and BCL11A in erythroblasts at 48 h after decitabine treatment.

To evaluate whether decitabine has superior HbF induction compared to HU, we treated primary human erythroblasts from healthy donors with either decitabine or HU and measured HbF by flow cytometry. Decitabine increased the frequency of F‐cells in a dose‐dependent manner in multiple donors (Figure 1A,B). We found that the maximum F‐cell response to decitabine was greater than to HU (Figure 1A,B).

The baseline and maximum levels of F‐cells induced by 1 µM decitabine, as well as the half‐maximal effective concentration (EC_50_) of decitabine, were quantified for four individual donors (Figure 1B). Although the baseline level of F‐cells varied across donors, ranging from 9.6% to 25.4%, decitabine increased F‐cells to between 71.2% and 84.9%. The EC_50_ values for decitabine ranged from 0.06 to 0.27 µM.

We further quantified the proportion of Hb variants after treatment. Both HU and decitabine increased HbF protein by approximately two‐fold in the primary erythroblasts of two donors (Figure 1C). Although both HU and decitabine achieved a similar magnitude of HbF protein induction, decitabine appears to promote HbF expression in a pan‐cellular manner, as evidenced by the higher F‐cell counts (Figure 1A,B).

To confirm the molecular mechanism that underlies the upregulation of HbF, we evaluated the expression of DNMT1 and BCL11A, both important regulators of HbF expression. The immunoblot revealed a reduction in DNMT1 expression with increasing concentrations of decitabine, while BCL11A protein abundance did not appear to be affected in the same way (Figure 1D).

### 3.2 Decitabine Administration Does Not Affect Body Weight Gain in Townes Mice

**FIGURE 2:**
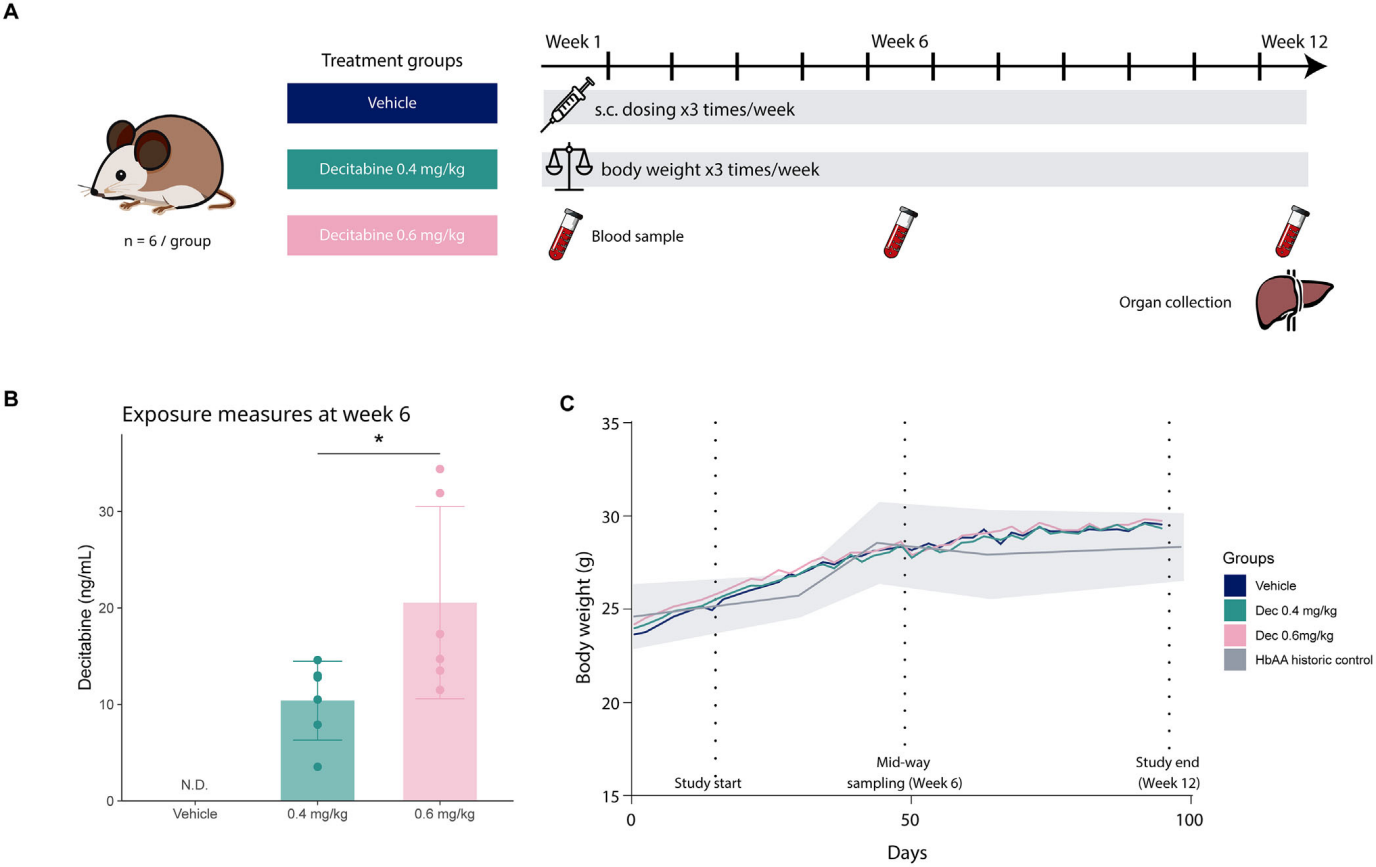
In vivo study design, exposure and growth. (A) Schematic of the in vivo experimental design depicting the treatment groups, dosing regimen, and timeline of the study. Townes SCD mice were divided into three treatment groups: vehicle (control), decitabine 0.4, and 0.6 mg/kg. Each group consisted of three male and three female mice. Treatments were administered subcutaneously three times a week, body weight was monitored thrice weekly and blood samples were collected at the beginning, midpoint, and end of the study, with organ collection conducted at the study's conclusion. (B) Decitabine exposure at Week 6 (ng/mL) in plasma; bars show the mean ± SD. (C) Body weight of the study mice over the study period. Lines represent the average body weight for each group: vehicle (control), decitabine 0.4 mg/kg, decitabine 0.6 mg/kg, and HbAA historic control 53; the shaded area indicates the SD for the HbAA controls. N.D. = not detectable. *n *= 6/group, *p < 0.05.

To study the translatability between human and animal systems, we conducted a study using the Townes mouse model of SCD [46, 50]. Mice aged 4–5 weeks were divided into three treatment groups: a vehicle group and two decitabine dose groups (0.4 and 0.6 mg/kg), administered subcutaneously (s.c.) thrice‐weekly (Figure 2A). To confirm exposure levels, plasma samples collected 45 min after the dose was administered on Day 36 (Week 6) were analysed. Vehicle‐treated mice had no detectable (N.D.) levels, while the 0.4 mg/kg dose group had a mean exposure of 45.4 nM, and the 0.6 mg/kg dose group had 89.8 nM (Figure 2B). Throughout the study period, the average body weight of SCD mice in all groups followed a general trend of increase. The body weight trajectories were comparable to those of untreated historic HbAA controls [53], indicating that decitabine, at the administered doses, did not cause discernible adverse effects on the mice's growth (Figure 2C).

### 3.3 Decitabine Treatment Influences Haematological Parameters in Townes Mice

**FIGURE 3:**
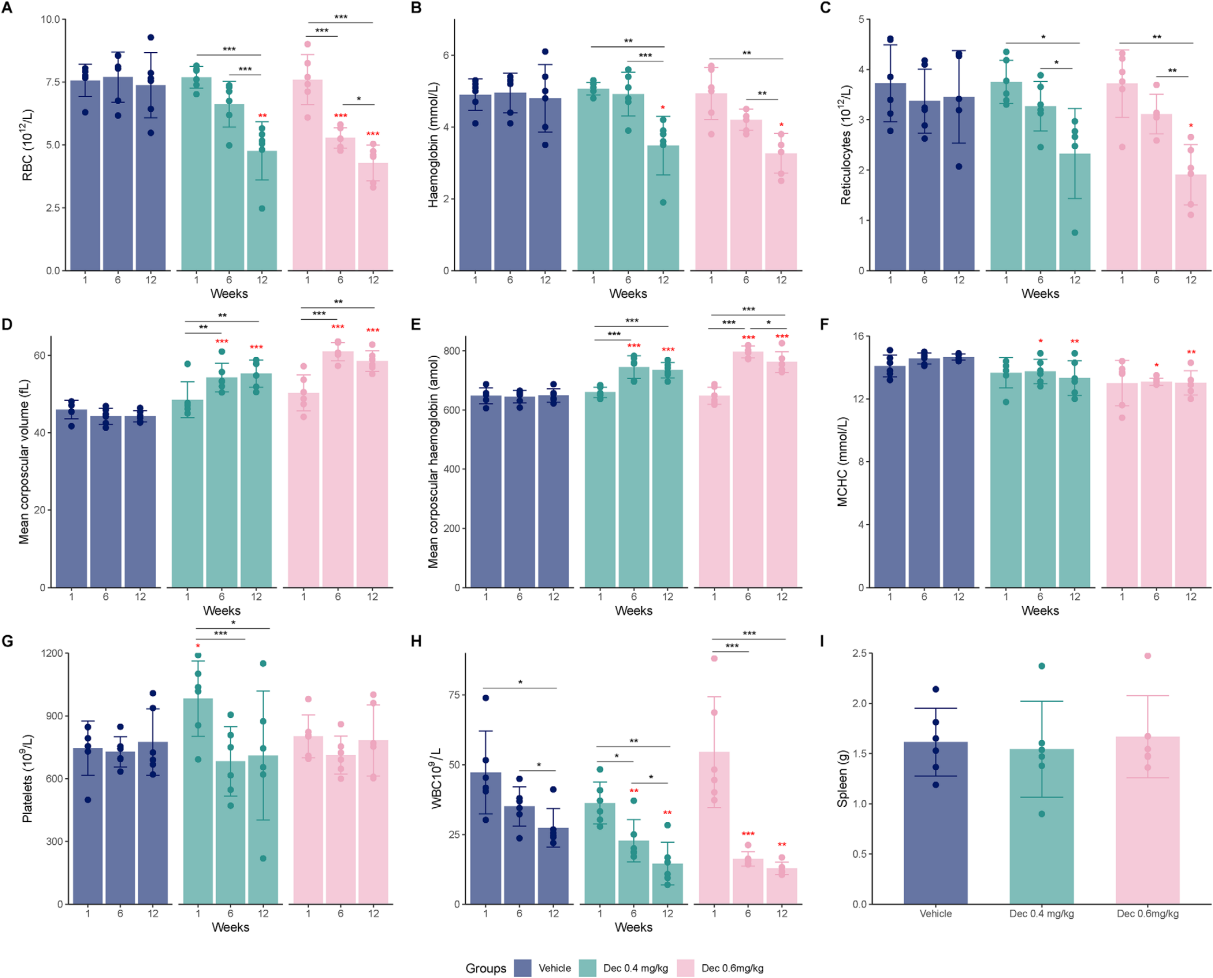
Impact of decitabine treatment on haematological parameters and spleen size in HbSS Townes mice. Each figure represents the three study groups: vehicle control, decitabine 0.4 and 0.6 mg/kg. Haematological parameters were assessed at the beginning of the study (Week 1), mid‐way (Week 6) and end of the study (Week 12). (A) Red blood cell count (RBC, 10^12^/L), (B) Haemoglobin concentration (Hb, mmol/L), (C) Reticulocyte counts (10^12^/L), (D) Mean corpuscular volume (MCV, fL), (E) Mean corpuscular haemoglobin (MCH, amol), (F) Mean corpuscular haemoglobin concentration (MCHC, mmol/L), (G) Platelet count (PLT, 10^9^/L), (H) White blood cell count (WBC, 10^9^/L), and (I) Spleen weight (g). Error bars indicate standard deviation. Data points represent individual mice. *p < 0.05, **p < 0.01 and ***p < 0.001 in black comparisons over time within the same group and in red compared to the vehicle group for the same timepoint, indicating statistical significance.

To assess the impact on haematological parameters, blood samples were obtained before dosing started (Week 1), midway through the study (Week 6), and upon study completion (Week 12). Initially, RBC counts between groups were comparable (mean ± SD; 7.57 ± 0.64 for vehicle, 7.69 ± 0.43 for 0.4 mg/kg and 7.60 ± 1.00 for 0.6 mg/kg). However, a notable decrease was observed in the decitabine‐treated groups, with the lowest levels at study end (7.38 ± 1.30 for vehicle, 4.76 ± 1.61 for 0.4 mg/kg and 4.29 ± 0.71 for 0.6 mg/kg) (Figure 3A). This decrease was also reflected in Hb concentration (Figure 3B) and haematocrit levels (Figure S1A). Furthermore, the observed decrease in reticulocyte counts in treated groups, which account for 50% of total RBCs, may indicate improved erythrocyte stability and reduced haemolysis (Figure 3C). MCV and MCH increased significantly in both treatment groups throughout the study (Figure 3D,E). MCHC was the least affected, but still, compared to the vehicle group, a significant decrease was observed for the two treated groups over time (Figure 3F). RDW‐SD increased in the treated groups compared to the vehicle group (Figure S1B). PLTs displayed high variability across groups and time points, with no statistically significant differences observed between groups except a higher baseline level for the 0.4 mg/kg group (Figure 3G). WBC counts decreased in all groups over time, suggesting a treatment‐independent effect on leukocyte counts (Figure 3H). Spleen weight at the conclusion of the study did not display significant differences, aligning with the characteristic splenomegaly observed in Townes mice [51] (Figure 3I).

To evaluate effects on erythropoiesis, plasma erythropoietin (EPO) levels were assessed. Vehicle‐treated mice showed significantly elevated EPO levels compared to C57Bl6 wild‐type controls, reflecting the ongoing and elevated erythropoiesis in SCD (Figure S1C). Meanwhile, decitabine treatment resulted in an upward trend in EPO levels, particularly driven by three mice that presented with low RBC and reticulocyte counts at termination (Figure S1D).

### 3.4 Decitabine Treatment Significantly Elevates HbF Levels and F‐Cells in Townes Mice

**FIGURE 4:**
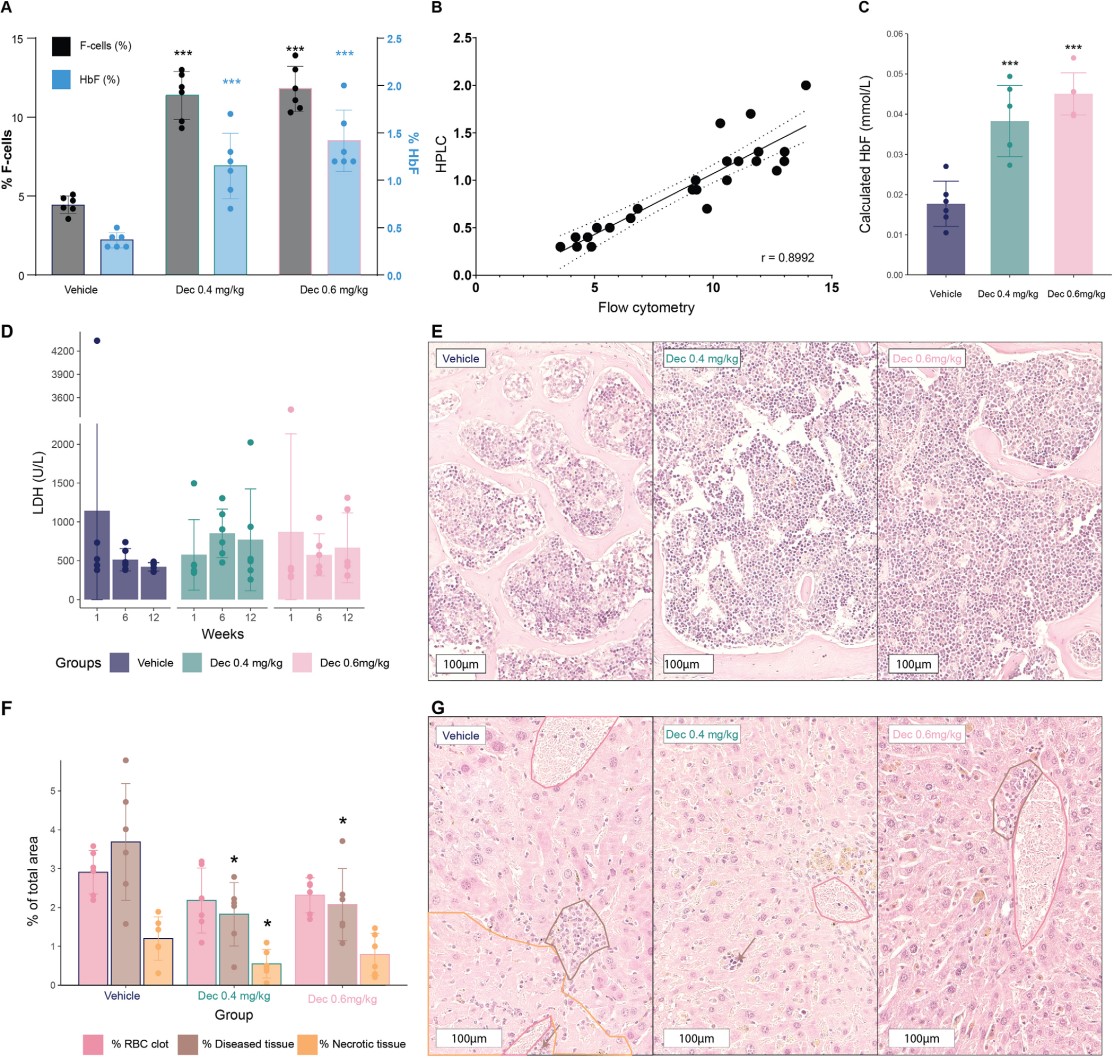
Foetal haemoglobin (HbF) induction post‐treatment with decitabine at Week 12 and effects of decitabine treatment on LDH, sternum and liver histology in HbSS Townes mice. (A) The left *y*‐axis (in black) shows the percentage of F‐cells (cells containing HbF) by flow cytometry, right *y*‐axis (in blue) shows the percentage of HbF protein levels by HPLC. (B) Correlation between HbF levels measured by HPLC and flow cytometry, displayed with its correlation coefficient (*r* = 0.9). (C) HbF levels calculated from HPLC data. (D) Lactate dehydrogenase (LDH) levels (U/L) for the three treatment groups (vehicle, decitabine 0.4, and 0.6 mg/kg) were measured at Weeks 1, 6 and 12. (E) Representative H&E histological sections of the sternum at 20× magnification for each treatment group. (F) Quantitative analysis of histological features classified by the liver classifier algorithm, affected by RBC clot, diseased tissue and necrotic tissue, presented as a percentage of the total area for each liver section from mice in each treatment group. (G) Representative H&E stained histological sections of the liver from each treatment group at 20× magnification with examples of RBC clots marked in pink, diseased tissue in brown and necrotic tissue in orange. Data points represent individual mice, with mean values indicated by bars and SD by error bars. *p < 0.05, ***p < 0.001 indicates significant differences from vehicle control. Scale bars in (E) and (G) indicate 100 µm.

Blood samples were obtained to examine HbF protein levels by HPLC, represented as a percentage of total Hb, and F‐cells by flow cytometry, represented as a percentage of the total RBC population positive for HbF, to evaluate the impact of decitabine in vivo. HbF was significantly elevated in both treatment groups compared to the control group, with the highest response observed at 12 weeks (Figure 4A). At Week 6, an intermediate response was observed, with mean F‐cell levels of 8.8% in the 0.4 mg/kg group and 9.3% in the 0.6 mg/kg group. At the study's conclusion, F‐cells were significantly higher in both the 0.4 mg/kg (11.4 ± 1.5%) and 0.6 mg/kg (11.8 ± 1.4%) groups compared to the vehicle group (4.5 ± 0.6%) (Figure 4A, in black). A similar dose‐dependent increase in HbF was observed by HPLC, with the high‐dose group showing a percentage of HbF nearly triple that of the control group (vehicle: 0.4 ± 0.1%, 0.4 mg/kg: 1.2 ± 0.3%, 0.6 mg/kg: 1.4 ± 0.3%) (Figure 4A, in blue).

A comparison of the two quantitation methods showed a strong positive correlation between HbF percentages from flow cytometry and HPLC (Figure 4B), with a correlation coefficient (*r*) of 0.899, corroborating the reliability of these independent measurement techniques.

We also calculated the molar HbF concentration based on Hb and HPLC measures and observed an increase in the treated groups. The mean HbF concentrations at the end of the study for the vehicle, 0.4 and 0.6 mg/kg groups were 0.02 ± 0.01, 0.04 ± 0.01 and 0.05 ± 0.01 mmol/L, respectively, indicating statistically significant increases following decitabine treatment (Figure 4C).

### 3.5 Effects of Decitabine on Haemolysis, Liver Health and Bone Marrow

To study the impact of treatment, we evaluated various biomarkers to monitor changes in disease state. LDH, a marker for haemolysis, remained unchanged throughout the study in all groups (Figure 4D). Markers of liver health, including AST, ALT, ALP and bilirubin, showed no differences between groups *(data not shown)*.

Examination of sternal bone marrow histopathology showed no differences among groups with regard to morphology. Sickle cells were present in all samples, and varying degrees of pigment accumulation were observed, most likely due to erythrocyte degradation. Yet, no apparent abnormalities in bone marrow cellularity were observed (Figure 4E). Notably, while we observed elevated numbers of megakaryocytes in the Townes sickle cell mice, consistent with active erythropoiesis, no significant differences were detected between the control and decitabine‐treated groups (Figure S1E).

Liver tissue damage was assessed using an algorithm trained to classify liver tissue in H&E‐stained liver sections from Townes mice [53]. Briefly, tissue damage was quantitatively assessed based on the proportion of the area corresponding to four distinct histological categories: normal liver tissue, RBC clots, necrotic tissue (tissue with infarcted areas), and diseased liver tissue, which included inflammatory cells, bile duct proliferation, and other tissue irregularities not found in healthy liver. Overall, at the end of the study, there was less tissue damage in the decitabine‐treated groups compared to the vehicle group; treated livers showed a significantly lower area of diseased tissue in both groups and necrotic tissue in the 0.4 mg/kg group (Figure 4F). Representative images of liver tissue are displayed in Figure 4G. While no differences in circulating biomarkers were observed between the groups, the reduction in liver tissue damage may indicate beneficial effects of decitabine on the treated mice.

## 4 Discussion

Robust preclinical models are needed to facilitate the development of new therapies for SCD. Given that decitabine is known to have HbF‐inducing properties [11, 13, 14, 16], we investigated its potential to increase HbF in both in vitro and in vivo preclinical models.

Our findings from human erythroblasts indicate that decitabine is more effective in elevating HbF levels compared to HU at the highest tolerated dose in vitro, both in terms of the increased percentage of F‐cells and total protein. HU‐treated RBCs are known to exhibit a pancellular distribution of HbF [57], and our data suggest a similar distribution in erythroblasts treated with decitabine. However, it is important to consider the limitations of our in vitro study, as continuous exposure to decitabine is not likely to accurately mimic in vivo conditions. The fast elimination of decitabine in vivo (in mice, intravenous half‐life is approximately 30 min [58], and less than 1 h given orally [36, 58]) indicates that steady‐state stable plasma levels are not achieved. Additionally, in our experiments, we observed that 10 µM decitabine reduced cell viability, while previous studies indicated that 0.5 µM was non‐toxic, but 1 µM could cause cellular stress [41]. Although our study did not specifically look for signs or markers of toxicity, this underscores the need to explore the effects of decitabine at different concentrations, given that the therapeutic index of decitabine appears to be relatively narrow in mice [37].

Decitabine induces DNA hypomethylation by directly incorporating into replicating DNA and inhibiting DNA methylation by DNMT1 depletion, leading to the reactivation of silenced genes and differentiation in cancer cells [22, 59]. We confirmed that HbF induction by decitabine in erythroblasts is associated with DNMT1 inhibition. Our data did not clearly show any such effect on BCL11A, a well‐characterized HbF repressor, indicating that BCL11A may not, or only to a lesser extent, be involved in this process.

The in vivo study in Townes mice corroborates the potential of decitabine as an HbF inducer in SCD, as expected given the published preclinical and clinical data [29, 30, 31, 32, 33, 34, 35, 37]. Mice treated with decitabine (0.4 or 0.6 mg/kg) thrice weekly for 12 weeks displayed no adverse effects on growth, with weight gain comparable to that of literature HbAA controls [53] and the control group. Both doses were well tolerated as far as our observations showed, with no apparent effect on physical appearance, body weight or activity.

While Townes mice have low baseline HbF levels [48] compared to patients with SCD, a clear dose‐dependent increase in HbF levels was observed in the treated mice. The highest response was observed by Week 12, with HbF protein levels nearly tripling in percentage in the higher dose group compared to the vehicle group. This increase was also seen in F‐cells in the same pattern, but at higher percentages. Moreover, we observed a strong correlation between measurements of both F‐cells and HbF protein levels. Additionally, the calculated HbF concentration derived from Hb and HPLC measures (reflecting total HbF blood content) showed an increase in HbF in the treated groups, despite an overall decrease in Hb levels.

Our study shows that a duration of 6 weeks is not sufficient to reach the full potential of either dose, and since we have not studied longer durations, we cannot say whether a plateau was reached by 12 weeks. Additionally, it is possible that less frequent dosing might yield similar results, since once decitabine is phosphorylated intracellularly during the S‐phase, it cannot leave the cell, potentially leading to accumulation or sustained efficacy even after the drug is cleared from plasma [22]. Due to its mechanism of action with incorporation into DNA, perhaps the effect may not depend on steady‐state stable exposure but rather on achieving sufficiently high peak exposures. Finally, it is important to consider the dose‐dependency of the observed effects; while the current study demonstrates a dose‐dependent increase in HbF levels, it remains uncertain whether further escalation of doses would yield significantly greater outcomes. Also, there is likely to be an upper limit to the tolerable dose, as suggested by existing literature [37]. Future studies should explore additional and longer dosing regimens to maximize HbF induction and aim for better tolerability by adjusting the dosing frequency.

Our observations of changes in haematological parameters provide insights into the influence of decitabine on erythropoiesis. While some parameters, such as lower total RBC, Hb levels and haematocrit post‐treatment, may suggest a myelosuppressive side effect, the significant rise in MCH indicates effective erythropoiesis stimulated by increased HbF levels. Although an increase in MCV was observed, SCD patients are generally microcytic, and in that context, increasing MCV may be beneficial. In Townes mice, this increase adds to the existing macrocytosis of the HbSS phenotype but could indicate increased hydration levels of RBCs. Preventing cellular dehydration and modifying intracellular water and solute content of sickle erythrocytes is a promising strategy in preventing complications of SCD [60], as seen with HU treatment, which also increases MCV [61, 62].

While the decrease in reticulocyte counts in the treatment groups may indicate a myelosuppressive effect, it could also potentially indicate an amelioration of the regenerative anaemia. Although further investigation of the latter hypothesis is warranted, the observation of decreasing total RBC counts and an increase in RDW‐SD does not immediately support it.

Furthermore, we observed elevated EPO levels in HbSS mice, aligning with literature reports [63]. There was a trend towards further increased EPO levels in decitabine‐treated mice, suggesting an increased effort to produce RBCs, albeit with significant variability between mice. We found that mice exhibiting particularly elevated EPO levels coincided with those having the lowest RBC counts. As mice age and become more fragile, we hypothesize that the accumulated physiological stress may lead to fluctuating complete blood counts. We have previously observed declines in RBC in untreated HbSS Townes mice as they age [53]. Thus, elevated EPO levels and reduced RBC counts in specific mice could be indicative of disease manifestations such as VOCs or haemolysis, independently of treatment.

WBC counts decreased across all groups, hinting at a treatment‐independent effect. Previous studies have reported similar decreases, attributing this to hypoxic conditions and VOC [51]. Long‐term follow‐up of Townes mice showed considerable variability in WBC counts over time [53], although no statistically significant decline was observed.

PLTs showed high variability without clearly discernible patterns related to treatment, indicating a potentially isolated effect of the drug on erythropoiesis rather than on thrombopoiesis. To further investigate whether there was a shift in myeloid commitment trajectories, we assessed megakaryocyte numbers in sternal bone marrow. Megakaryocyte counts were elevated in all mice across groups compared to available literature on wild‐type mice [64]. Overall, while our assessment of megakaryocyte numbers indicates active erythropoiesis, the data do not conclusively demonstrate a significant shift in myeloid commitment trajectories among the treatment groups, as the variation within groups exceeded any discernible effect.

The parallel assessment of disease markers and histological examination of organs such as the liver and sternum provides additional insights into the systemic effects of decitabine. Histopathological examination of sternal bone marrow revealed no significant morphological changes across different treatment groups. The presence of sickle cells and varying degrees of pigment accumulation, probably due to erythrocyte degradation, was noted uniformly, indicating that treatment did not adversely affect bone marrow cellularity or morphology. Although markers of haemolysis, like LDH levels, did not show significant changes in response to treatment, there were changes in liver damage assessed from HE‐stained liver slides. We utilized an algorithm to classify histological features in liver tissue, such as RBC clots, necrotic tissue (indicative of a more chronic lesion) and diseased liver tissue (indicative of a more acute lesion). We observed a decreased fraction of necrotic and diseased liver tissue in treated animals, suggesting a protective effect of decitabine, pointing to reduced ischaemic events and improved organ integrity.

Although the Townes model resembles human SCD in many ways, and our findings from in vitro and in vivo models offer consistent and promising results, they are subject to certain limitations intrinsic to using mouse models for research in SCD and HbF induction. Significant challenges are caused by discrepancies in globin gene regulation and configurations between humans and the transgenic mouse models. As recently shown, the genomic structures in these mouse models differ significantly from human configurations, lacking certain key distal regulatory DNA elements crucial in controlling β‐like globin gene expression [47]. Moreover, unlike humans, the wild‐type mouse globin cluster contains only ɛ and adult globin genes, lacking the ortholog gene encoding for foetal globin [42]. This complicates the direct translation of our findings and could potentially lead to an underestimation of the potential of decitabine in a clinical context. Nevertheless, insights from clinical data on other HbF‐inducing therapies may enrich our understanding of how these preclinical models can be applied.

In summary, our study supports the potential of decitabine as a potent inducer of HbF while also demonstrating protective effects on liver tissue integrity in a mouse model. Further optimization of the study design, including dosing regimen and duration, may enhance these effects. While the translational relevance of our findings from mouse models to human disease remains to be fully established, the consistency observed across in vitro and in vivo models is promising and informative.

## Author Contributions

**Ariadna Carol Illa**: conceptualization, data curation, formal analysis, investigation, methodology, software, visualization, writing – original draft, writing – review and editing. **Desmond Wai Loon Chin**: investigation, methodology, writing – review and editing. **Martha Clark**: investigation, methodology, writing – review and editing. **Jesper Petersen**: investigation, methodology, writing – review and editing. **Søren Skov**: funding acquisition, supervision, writing – review and editing. **Andreas Glenthøj**: supervision, writing – review and editing. **Carsten Dan Ley**: conceptualization, funding acquisition, supervision, writing – review and editing.

## Conflicts of Interest

Ariadna Carol Illa, Desmond Wai Loon Chin, Martha Clark, and Carsten Dan Ley are employees and/or shareholders of Novo Nordisk A/S. Søren Skov has received consultancy fees from Novo Nordisk and research support from DSM‐Firmenich and Novo Nordisk. Andreas Glenthøj has received consultancy and advisory board fees from Agios, Novo Nordisk, Pharmacosmos, and Vertex Pharmaceuticals, as well as research support from Agios, Bristol Myers Squibb, Novo Nordisk, Saniona, and Sanofi. Jesper Petersen has nothing to disclose.

## Supporting information




**Supporting File 1**: jha270120‐sup‐0001‐SuppMat.pdf

## Data Availability

The data that support the findings of this study are available from the corresponding author upon reasonable request.
