# Supplementary material for: Decitabine‐Driven Foetal Haemoglobin Induction in Townes Mice and Human Erythroblasts
Source: EJHaem. 2025 Aug 4;6(4):e70120. doi: 10.1002/jha2.70120 (PMC12320723; doi:10.1002/jha2.70120)
Supplement: Supplementary file 1 — Supporting File 1: jha270120‐sup‐0001‐SuppMat.pdf [file JHA2-6-e70120-s001.pdf]

Decitabine-Driven Foetal Haemoglobin Induction in Townes Mice and Human Erythroblasts

Ariadna Carol Illa[1,2*], Desmond Wai Loon Chin[3], Martha Clark[3], Jesper Petersen[4], Søren Skov[2], Andreas Glenthøj[4,5], Carsten Dan Ley[1]

[1] Rare Disease Research, Global Drug Discovery, Novo Nordisk A/S, Måløv, Denmark

[2] Department of Veterinary and Animal Sciences, Faculty of Health and Medical Sciences, University of Copenhagen, Copenhagen, Denmark

[3] Rare Disease Research, Global Drug Discovery, Novo Nordisk Research & Development US, Inc., Boston, USA

[4] Danish Red Blood Cell Center, Department of Hematology, Copenhagen University Hospital – Rigshospitalet, Copenhagen, Denmark

[5]Department of Clinical Medicine, University of Copenhagen, Copenhagen, Denmark

Correspondence: Ariadna Carol Illa (ezai@novonordisk.com), ORCID-ID: 0000-0002-6925-4493

Supplementary data

Supplementary figure 1

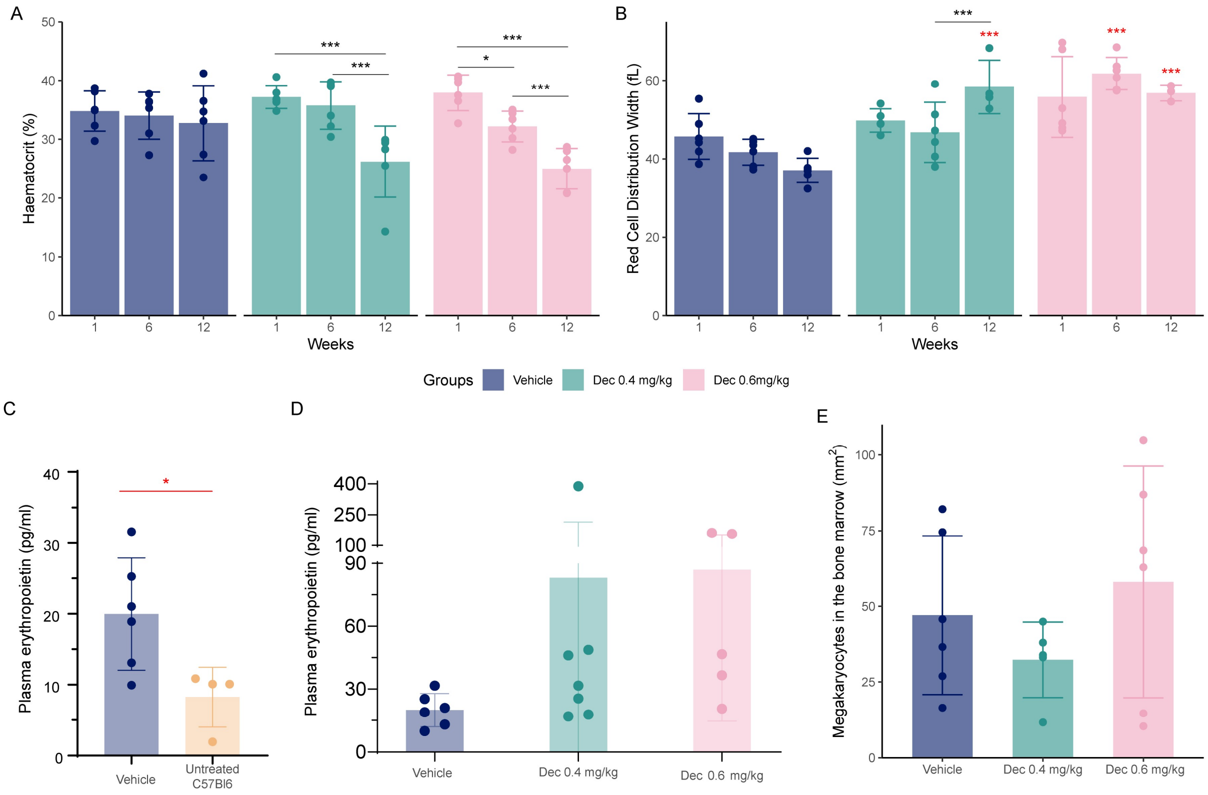

Supplemental Figure 1. Effects of decitabine treatment on certain haematology-related parameters in HbSS Townes mice. (A) Haematocrit levels (%) and (B) Red cell distribution width (RDW-SD, fL) measured at weeks 1, 6 and 12 in treatment groups: vehicle, decitabine 0.4 mg/kg, and decitabine 0.6 mg/kg. (C-D) Plasma erythropoietin levels (pg/ml) assessed at the study conclusion, with panel C showing data from the vehicle group and untreated C57BL/6 mice, and panel D including data for vehicle and decitabine-treated groups. (E) Mean megakaryocyte numbers in the bone marrow (cells/mm^2). Data are presented as individual data points with bars representing the mean ± SD. Statistical significance is indicated by asterisks: *$p < 0.05$, **$p < 0.01$, ***$p < 0.001$, comparing measurements over time within the same group (black) and treated groups vs. the vehicle group (red).

Supplementary methods

Erythropoietin ELISA

To quantify mouse erythropoietin (EPO) levels, the Mouse EPO ELISA Kit (#EM28RB, Invitrogen) was utilized according to the manufacturer's instructions. Terminal plasma samples were diluted 1:1 with 1X Assay Diluent prior to analysis.

Bone marrow megakaryocytes assessment

Sternum sections of 3 μm thickness were stained with haematoxylin and eosin (H&E) to visualize megakaryocyte morphology and quantity. The assessment was conducted in a blinded manner, and the number of megakaryocytes was quantified per square millimetre (cells/mm^2) from representative fields across each treatment group using VISIOPHARM software.
